# Mitigating the impact of adolescence isolation on the development of social anxiety: A potential role for oxytocin

**DOI:** 10.3389/fnbeh.2022.1038236

**Published:** 2022-10-13

**Authors:** Morgan P. Johnston, Matthew J. Wanat

**Affiliations:** Department of Neuroscience, Developmental, and Regenerative Biology, Neurosciences Institute, Brain Health Consortium, University of Texas San Antonio, San Antonio, TX, United States

**Keywords:** social anxiety, adolescence, oxytocin, sex differences, sex hormones, isolation

## Abstract

Exposure to isolation can lead to the development of social anxiety disorder (SAD), which affects 13% of Americans. There are sex differences in the prevalence of anxiety disorders, as women experience higher rates of SAD relative to men. Importantly, isolation experienced during adolescence increases the likelihood of developing SAD in adulthood. Unfortunately, the current treatments for SAD are only effective in 50–65% of patients. As such, it is critical to identify therapeutic targets for the treatment and prevention of SAD, particularly in women. Here, we discuss the links between childhood isolation and adulthood SAD. Next, we examine the preclinical models used to study the impact of isolation on social anxiety-like behaviors in rodents. Increasing evidence from both clinical and pre-clinical studies suggests oxytocin signaling is a potential target to modify social anxiety-like behaviors. We present the evidence that sex hormones influence the oxytocin system. Finally, we highlight future directions for both clinical and pre-clinical studies to further evaluate the efficacy of oxytocin as a treatment for isolation-induced SAD.

## Introduction

Social isolation can lead to the development of anxiety disorders (Muller et al., 2019; Nelemans et al., 2019; Hards et al., 2021). The effects of isolation on mental health can be influenced by both age and sex (Wright et al., 2009; Craner et al., 2022; Culpin et al., 2022; Garcia-Fernandez et al., 2022; Jiang et al., 2022; Smith et al., 2022). Understanding the impact of social isolation has become increasingly vital due to the COVID-19 pandemic. Lockdowns and school closures early on in the pandemic resulted in many adolescents having less face-to-face interactions with their peers. Since 2019 there has been an increase in children reporting feelings of isolation and loneliness (Hards et al., 2021; Centers for Disease Control and Prevention, 2022). Importantly, isolation in childhood can lead to the development of mental health disorders, including social anxiety disorder (SAD) (Muller et al., 2019; Hards et al., 2021; Craner et al., 2022). Women and adolescent girls consistently report higher rates of stress, anxiety, and SAD relative to their male counterparts, which has been exacerbated by the COVID-19 pandemic (Boson et al., 2022; Garcia-Fernandez et al., 2022; Jiang et al., 2022; Smith et al., 2022; Whitten et al., 2022). As such, it is critical to identify therapeutic targets for the treatment and prevention of isolation-induced SAD, particularly in women. In this mini-review, we will examine the links between childhood isolation and adulthood SAD. Next, we will discuss pre-clinical evidence that oxytocin may be involved in isolation-induced SAD. Finally, we highlight the need for future clinical and pre-clinical research to further evaluate the potential of oxytocin as a treatment for SAD.

## Social anxiety disorder

### Clinical data

Social anxiety disorder (SAD) affects 13% of Americans and is a class of anxiety disorders characterized by a fear of social situations and possible scrutiny by others (American Psychiatric Association, 2013; Leichsenring and Leweke, 2017). Furthermore, exposure to social situations can induce or enhance anxious behaviors in individuals with SAD (American Psychiatric Association, 2013). However, the impact of anxiety disorders like SAD goes beyond an avoidance of social situations. In adults, SAD has been linked to a higher likelihood of persistent substance use, severe depression symptoms, and a reduced tendency to seek professional help (Beesdo et al., 2007; Leichsenring and Leweke, 2017; Elling et al., 2022). Additionally, SAD is more common in women than men in cultures around the world (McLean et al., 2011; Asher et al., 2017; Garcia-Fernandez et al., 2022; Jiang et al., 2022; Mngoma and Ayonrinde, 2022; Smith et al., 2022; Whitten et al., 2022). While a number of social theories have been presented as contributors to this sex difference, it is also thought to be due, in part, to differences in estradiol and androgen levels (Bahrami and Yousefi, 2011; McLean et al., 2011; Altemus et al., 2014; Gerdes et al., 2021; Sheng et al., 2021; Christiansen et al., 2022; Dark et al., 2022; Nouri et al., 2022). In particular, clinical studies have found that anxiety is higher in women when estradiol levels are elevated and that androgens have an anxiolytic effect in men (Graham and Shin, 2018; Sheng et al., 2021).

In addition to sex, the age at which one experiences isolation can impact how it affects their mental health (Wright et al., 2009; Lopez-Patton et al., 2016; Muller et al., 2019; Hards et al., 2021; Culpin et al., 2022). Feelings of isolation and emotional neglect in adolescence are risk factors for the development of SAD in adulthood (Muller et al., 2019; Hards et al., 2021). Examining how childhood isolation can lead to adulthood SAD has become increasingly relevant due to the COVID-19 pandemic, which saw many school closures and cancelations of events where children would typically have face-to-face interactions with their peers. Isolation from peers and parental figures during adolescence has been linked to increased substance use, emotional distress, depression, anxiety, and symptoms of SAD in adulthood (Wright et al., 2009; Lopez-Patton et al., 2016; Culpin et al., 2022). The percentage of adolescents reporting a sense of chronic loneliness has increased from 22% prior to the COVID-19 pandemic to 53% in 2021 (Hards et al., 2021; Centers for Disease Control and Prevention, 2022). Thus, it is likely that we will continue to see prolonged mental health consequences due to the isolation experienced during the COVID-19 pandemic. It is vital to identify strategies and therapeutic targets to reverse, or potentially prevent, the impact of isolation on mental health disorders. To this end, pre-clinical rodent studies have examined the link between isolation and SAD-like behaviors.

### Rodent models of isolation and social anxiety

Rodent studies examining isolation are typically performed by housing rodents alone for a period of time (Krimberg et al., 2022). Many studies investigating the effects of early-life isolation will separate a pup from the mother for 15 min – 6 h per day for several days in a row (Lukas et al., 2010; Oreland et al., 2010; Farrell et al., 2016; Baracz et al., 2022; Tran et al., 2022). Maternal separation can produce a number of behavioral alterations. For example, pups isolated from their mothers will vocalize less when reunited with the mother (Zimmerberg et al., 2003; Wohr and Schwarting, 2008). Additionally, maternally isolated pups exhibit deficits in exploration when placed in a novel context (Modlinska et al., 2018; Kambali et al., 2019). While these studies provide valuable insight to the effects of isolation from parental figures, they do not address how isolation from peers impacts behavior. To better model this, some studies remove adolescent rodents from their peers after weaning (Makinodan et al., 2012; Oliveira et al., 2019; Yamamuro et al., 2020; Park et al., 2021; Musardo et al., 2022; Shan et al., 2022).

Rodents can exhibit a number of “anxiety-like” behaviors, such as auto-grooming, burrowing, avoidance, freezing, and darting (Lezak et al., 2017; Oliveira et al., 2019; Krimberg et al., 2022). These behaviors are deemed “anxiety-like” because they resemble anxious behaviors in humans, but it is important to acknowledge that we cannot infer the mental state of a rodent (Lezak et al., 2017). Common assays of anxiety-like behaviors include the elevated plus maze and open field tests (Lezak et al., 2017). In these tests, spending more time in the closed portion of the apparatus or near the walls of the enclosure is associated with an anxiety-like phenotype (Lezak et al., 2017). Using these assays and others, studies have found that female rodents display more anxiety-like behaviors than male rodents (Bishnoi et al., 2021). These anxiety-like behaviors are positively correlated with estradiol levels, which fluctuate throughout the estrous cycle (Ajayi and Akhigbe, 2020; Pidoplichko et al., 2021). This parallels findings in humans, which supports the use of rodent models to examine heightened instances of anxiety in females (Graham and Shin, 2018; Pidoplichko et al., 2021).

While the elevated plus maze and open field tests have been the basis for much anxiety-related research, they are not ideally suited to examine SAD-like behaviors because they lack a social component. One manner in which social anxiety-like behaviors are assessed is using a social interaction test (Lezak et al., 2017; Harro, 2018; Krimberg et al., 2022). In this test, two rodents are placed in a cage together and researchers observe how they interact with each other and measure behaviors such as aggression, avoidance, burrowing, sniffing, offensive grooming, and auto-grooming (Lezak et al., 2017; Harro, 2018). However, the behavior of one rodent may influence the behavior of the other, and thus it is difficult to measure individual levels of anxiety-like behavior (Harro, 2018). Because the animals are able to interact with each other freely, the social interaction test is considered a measure of direct social interaction (Huang et al., 2021). Tests such as a social lever pressing task and social preference test are used to measure indirect social interaction and avoid the issue of another rodent influencing the behavior of the experimental rodent (Toth and Neumann, 2013; Harro, 2018; Huang et al., 2021). By training rodents to press a lever to gain temporary access to a social partner, researchers are able to measure the rodent’s motivation to seek out social interactions (Solie et al., 2022). Social preference tests often utilize a three chambered cage where the rodent that is being tested is placed in the middle chamber and the two outside chambers will contain either another rodent or an object (Toth and Neumann, 2013). It is important to note that during this test the two rodents are unable to freely interact with each other due to a physical barrier (Toth and Neumann, 2013). Social preference tests are ideally suited to model SAD-like behaviors in rodents, as they provide a measure of the willingness of a rodent to interact with a conspecific versus an inanimate object (Toth and Neumann, 2013).

Isolation from peers during adolescence alters social behaviors (Oliveira et al., 2019; Huang et al., 2021; Baracz et al., 2022; Krimberg et al., 2022). For example, isolation reduces a rodent’s ability to distinguish between known and unknown juvenile rodents (Oliveira et al., 2019). A study utilizing both the social interaction and social preference tests in male mice found that post-weaning isolation decreased the amount of time a mouse spent interacting either directly or indirectly with an unfamiliar conspecific (Makinodan et al., 2012; Huang et al., 2021). This decrease in social interactions is unable to be rescued by reintroducing the mice to their peers (Makinodan et al., 2012). Additionally, isolation in adolescence can have long lasting consequences on behavior. Specifically, rats that experience isolation in adolescence display increased aggression toward conspecifics in adulthood (Oliveira et al., 2019; Baracz et al., 2022). Finally, isolation experienced in adulthood increases anxiety-like behaviors in rodents (Doremus et al., 2004; Zorzo et al., 2019; Evans et al., 2020). However, it is important to note that many of these studies were only performed in males. In one study using both sexes, females isolated during adolescence exhibited greater aggression toward juveniles relative to group housed females and isolated males (Oliveira et al., 2019). This highlights the need to study the impact of isolation on social and anxiety-like behaviors in both sexes. Collectively, these studies demonstrate that early-life isolation can lead to long-lasting behavioral consequences. Ultimately, there is a critical need to identify the neural systems involved in isolation-induced SAD.

## Oxytocin’s role in isolation-induced social deficits and anxiety-like behaviors

Oxytocin is a neuropeptide that is primarily synthesized in the hypothalamus and has been implicated in facilitating social interactions (Love, 2018). While oxytocin is perhaps best known for its roles in childbirth, nursing, and pair-bonding, it is also involved in a myriad of other behaviors such as social cognition and perception, mood, and harm avoidance (Bartz and Hollander, 2006; Love, 2014, 2018; Yoon and Kim, 2020; Rigney et al., 2022). Similar to humans, oxytocin in rodents is typically associated with pro-social behavior. For example, non-aggressive social touch increases oxytocin neuron activity, oxytocin release is necessary for social reward, and oxytocin is a key modulator of pair-bonding in rodents (Hung et al., 2017; Bosch and Young, 2018; Tang and Graham, 2020; Tang et al., 2020). Rats separated from their mothers experience deficits in social behavior which are associated with decreased oxytocin receptor binding and lower immunoreactive oxytocin levels (Lukas et al., 2010; Oreland et al., 2010). Additionally, antagonizing oxytocin receptors in juvenile rodents mimics the behavioral effects of isolation (Huang et al., 2021). These roles of oxytocin in social behaviors have made it the subject of many studies investigating potential therapeutic targets for SAD.

### Sex differences in oxytocin

Differences in sex hormones, such as estradiol and testosterone, are thought to be partially responsible for the sex differences in SAD (Graham and Shin, 2018; Pidoplichko et al., 2021; Sheng et al., 2021). Oxytocin and estradiol levels fluctuate with the estrous cycle, which is likely partially responsible for estrous-dependent changes in anxiety-like behavior (Zhang et al., 2008; Bertram et al., 2010; Ajayi and Akhigbe, 2020; Pidoplichko et al., 2021). Lower anxiety-like behaviors are associated with the estrus phase, during which estradiol and oxytocin levels peak (Bertram et al., 2010; Ajayi and Akhigbe, 2020; Pidoplichko et al., 2021). Endogenous increases in estradiol are also associated with elevated oxytocin receptor binding (Johnson, 1992). Furthermore, estradiol administration increases oxytocin secretion and activation of some estradiol receptor subtypes increases oxytocin peptide transcription (Brown et al., 2008; Acevedo-Rodriguez et al., 2015). Androgens may also influence anxiety-like behavior by increasing oxytocin production (Sheng et al., 2021). Collectively, these studies highlight the importance of examining sex hormones as biological variables when studying isolation-induced SAD.

Additional sex differences have been identified in the oxytocin system of rodents. For example, oxytocin receptor binding can vary by sex depending on the brain region being examined (Oliveira et al., 2019; Ross et al., 2019). Social isolation decreases oxytocin receptor binding in adult females but has no effect on oxytocin receptor binding in adult males (Ross et al., 2019). In juveniles, however, isolation decreases oxytocin receptor binding regardless of sex (Oliveira et al., 2019). These data indicate that the effect of isolation on the oxytocin system is both sex- and age-dependent.

## Oxytocin administration to normalize social anxiety disorder-like behaviors

### Rodent data

Rodent studies have found that global administration of an oxytocin receptor agonist decreases anxiety-like behaviors that arise from isolation (Krimberg et al., 2022). Increases in oxytocin are associated with decreased freezing responses during fear conditioning and reduced anxiety-like behavior (Viviani et al., 2011; Lee et al., 2017; Janecek and Dabrowska, 2019; Wahis et al., 2021). Additionally, oxytocin injections increase non-sexual social behavior in male rats, such as sniffing and grooming (Witt et al., 1992). Oxytocin administration also decreases blood pressure and cortisol levels, and these anxiolytic effects become more pronounced with repeated treatments (Uvnas-Moberg, 1997a,b, 1998a,b). However, there have been conflicting reports regarding oxytocin’s anxiolytic effects in juveniles. One study found that repeated oxytocin administration following a stress experience during adolescence prevented the development of anxiety-like behaviors in adulthood (Baracz et al., 2022). Yet another demonstrated that inhibiting oxytocin neurons during behavioral assays of anxiety reversed isolation-induced social deficits (Musardo et al., 2022). These data suggest that there may be a therapeutic benefit of oxytocin treatments, but more research is necessary to identify the ideal temporal window for maximum efficacy. Additionally, a growing body of literature suggests that oxytocin treatments may have different results depending upon the brain region administered and whether oxytocin is acting *via* neurons or astrocytes (Parent et al., 2008; Li et al., 2016, 2021; Havranek et al., 2017; Oliveira et al., 2019; Jang et al., 2021; Wahis et al., 2021; Musardo et al., 2022). Determining the cellular mechanisms by which oxytocin exerts its anxiolytic effects will be critical for its use in the development of efficacious treatments for isolation-induced SAD.

### Clinical data

SAD has been linked to alterations in the oxytocin system, such as genetic and epigenetic variations in the oxytocin receptor gene (Gottschalk and Domschke, 2018; Nelemans et al., 2019). However, these studies cannot determine whether the relationship between SAD and oxytocin is casual or correlative. Regardless, these studies highlight that the oxytocin system is a potential target to treat SAD symptoms. As such, studies have examined whether administering oxytocin in humans could be of therapeutic benefit to individuals with SAD. Administering intranasal oxytocin to adult men with SAD improves their perception of themselves and their confidence, but this effect does not generalize to all aspects of SAD (Guastella et al., 2009). Oxytocin administration also enhances social affiliation and cooperation in certain subsets of men with SAD (Fang et al., 2014). Furthermore, a clinical study where healthy adult men received intranasal oxytocin and/or social support during a stress test found that combining the two resulted in the lowest cortisol levels, highest self-reported calmness, and decreased anxiety (Bartz and Hollander, 2006). It is important to note that the effects of oxytocin may extend beyond reducing SAD symptoms, as human data has shown that oxytocin administration can alleviate anxiety and fear in non-social settings as well (Mitchell et al., 2015; Koch et al., 2016; Janecek and Dabrowska, 2019; Horta et al., 2020; Yoon and Kim, 2020). Individuals with SAD often experience comorbid anxiety disorders, which presents a challenge in treating these patients (Koyuncu et al., 2019). Thus, developing a therapeutic strategy that may improve both general anxiety symptoms and more specific SAD symptoms would be advantageous. Collectively, these data demonstrate that oxytocin may have a therapeutic benefit for individuals with SAD. However, due to potential side effects associated with manipulating oxytocin in adult women, such as uterine contractions and alterations to the menstrual cycle, clinical oxytocin studies have largely focused on adult men (Asher et al., 2017).

## Future directions

The current standard of treatment for patients with SAD, which includes cognitive behavioral therapy and selective serotonin reuptake inhibitors, are only effective in 50–65% of patients (Leichsenring and Leweke, 2017). This underscores the need for the development of new therapeutic targets. Pre-clinical studies have shown that isolation can lead to social deficits and decreased functioning of the oxytocin system (Lukas et al., 2010; Oreland et al., 2010; Makinodan et al., 2012; Oliveira et al., 2019; Huang et al., 2021; Baracz et al., 2022; Krimberg et al., 2022). Additionally, antagonizing oxytocin receptors can recapitulate the effects of isolation on behavior (Huang et al., 2021). Estradiol and androgens, as well as sex differences in the oxytocin system, likely play a role in sex differences in isolation-induced SAD (Johnson, 1992; Brown et al., 2008; Bertram et al., 2010; Acevedo-Rodriguez et al., 2015; Oliveira et al., 2019; Ross et al., 2019; Pidoplichko et al., 2021; Sheng et al., 2021). However, important gaps in knowledge remain. It will be vital for future pre-clinical research to examine how the timing of oxytocin treatment(s) following isolation influences social anxiety-like behavior. Additionally, much of our current literature on the impact of adolescent isolation on anxiety-like behaviors focuses on male rodents. More research is necessary to know whether isolation has a similar impact on females and if the effects of isolation may be dependent upon the estrous cycle. Furthermore, pre-clinical literature delineating the effects of oxytocin administration in specific brain regions and cell populations on isolation and social anxiety-like behaviors is necessary.

Human oxytocin administration studies have primarily focused on adult men. However, rodent studies indicate that oxytocin treatments during adolescence may be an effective preventative treatment for isolation-induced SAD. Thus, clinical studies on oxytocin administration in children are necessary to know if oxytocin treatments may be safe and effective for that population (Baracz et al., 2022). Future studies will also need to take into account endogenous fluctuations in oxytocin due to hormonal cycles or other life events, such as pregnancy and aging. Finally, it is important to acknowledge that the human studies referenced here used cis-gender women and men as subjects. Future research would benefit from examining whether gender-affirming hormone therapy may impact the effectiveness of oxytocin treatments. Specifically, because testosterone appears to have an anxiolytic effect, could taking exogenous testosterone increase the effectiveness of oxytocin treatments, and could exogenous estrogen have a blunting effect? Collectively, these future studies will be instrumental in the development of better treatments and preventative care for individuals with isolation-induced SAD.

## Author contributions

MJ wrote the first draft, MW assisted with edits. Both authors contributed to the article and approved the submitted version.
